# Genetic Dissection of Rice Ratooning Ability Using an Introgression Line Population and Substitution Mapping of a Pleiotropic Quantitative Trait Locus *qRA5*

**DOI:** 10.3390/plants11091134

**Published:** 2022-04-22

**Authors:** Hui Hu, Ruoyu Gao, Liping He, Famao Liang, Zhixin Li, Junying Xu, Longwei Yang, Chongrong Wang, Zhangyong Liu, Jianlong Xu, Xianjin Qiu

**Affiliations:** 1 Hubei Collaborative Innovation Center for Grain Industry, College of Agriculture, Yangtze University, Jingzhou 434025, China; 240925404huhui@sina.com (H.H.); 18107187537gry@sina.com (R.G.); he11253527@163.com (L.H.); lizhixin09@163.com (Z.L.); jyxu@yangtzeu.edu.cn (J.X.); ylwei1968@126.com (L.Y.); 2 Yichang Academy of Agricultural Sciences, Yichang 443004, China; liangfamao92@hotmail.com; 3 Guangdong Provincial Key Laboratory of New Technology in Rice Breeding, Rice Research Institute, Guangdong Academy of Agricultural Sciences, Guangzhou 510640, China; wangcr1980@163.com; 4 Institute of Crop Science, Chinese Academy of Agricultural Sciences, Beijing 100081, China; 5 Shenzhen Branch, Guangdong Laboratory for Lingnan Modern Agriculture, Agricultural Genomics Institute at Shenzhen, Chinese Academy of Agricultural Sciences, Shenzhen 518120, China

**Keywords:** rice ratooning ability, introgression line, quantitative trait locus, pleiotropic effect, substitution mapping

## Abstract

Ratooning ability is a key factor that influences ratoon rice yield, in the area where light and temperature are not enough for second season rice. In the present study, an introgression line population derived from Minghui 63 as the recipient parent and 02428 as the donor parent was developed, and a high-density bin map containing 4568 bins was constructed. Nine ratooning-ability-related traits were measured, including maximum tiller number, panicle number, and grain yield per plant in the first season and ratoon season, as well as three secondary traits, maximum tiller number ratio, panicle number ratio, and grain yield ratio. A total of 22 main-effect QTLs were identified and explained for 3.26–18.63% of the phenotypic variations in the introgression line population. Three genomic regions, including 14.12–14.65 Mb on chromosome 5, 4.64–5.76 Mb on chromosome 8, and 10.64–15.52 Mb on chromosome 11, were identified to simultaneously control different ratooning-ability-related traits. Among them, *qRA5* in the region of 14.12–14.65 Mb on chromosome 5 was validated for its pleiotropic effects on maximum tiller number and panicle number in the first season, as well as its maximum tiller number ratio, panicle number ratio, and grain yield ratio. Moreover, *qRA5* was independent of genetic background and delimited into a 311.16 kb region by a substitution mapping approach. These results will help us better understand the genetic basis of rice ratooning ability and provide a valuable gene resource for breeding high-yield ratoon rice varieties.

## 1. Introduction

As one of the important staple crops, rice is very important for humans worldwide, especially for people in Asia. In the last three decades, rice yield has sharply increased due to the use of the semi-dwarf gene and heterosis [1]. However, because of the explosive increase in the world’s population, the rice yield must be largely increased in the next half-century to meet the needs of the world’s population. This rice yield increase depends on a higher yield and more frequent harvests on the existing land [2]. Since the agricultural acreage is hard to increase, we have to increase rice production by improving the rice yield or cropping index per unit [3]. Due to the increasing cropping index, double-season rice could largely increase the total yield per unit. However, this double-season system requires a lot of fertilizer, water, and labor, resulting in large green-house gas emissions [3,4] and a high risk of yield loss because of cold-dew wind during the flowering stage in the second season. Ratoon rice is an alternative to second season rice in double-cropping areas where light and temperature are not enough for second season rice. It could also enhance the cropping index. Moreover, it requires fewer agricultural inputs and is environmentally friendly [5].

Ratooning is defined as regenerating new tillers after harvest [6], and the yield of ratoon rice largely depends on the ratooning ability. The ratooning ability is controlled by genetic components and is affected by many other factors, such as the growing conditions (light and temperature), production management, and growth duration [7]. To date, very few researchers have reported their findings on ratooning ability, and its genetic basis is still unknown. Tan et al. detected six quantitative trait loci (QTLs) for ratooning ability by a doubled haploid population [8]. Zheng et al. identified one QTL for ratooning ability and three QTLs for yield-related traits in the ratoon season [9]. Yang et al. identified 36 QTLs for yield-related traits in the first season and ratoon season by a recombinant inbred line population [10]. Among them, two QTLs were detected for ratooning ability. Three QTLs on chromosomes 7 and 8 were detected for ratooning ability by a recombinant inbred line population [11]. Using advanced backcross lines derived from *Oryza sativa* and *Oryza rufipogon*, a QTL named *qRAT5* was detected between InDel3 and RM249 on chromosome 5 [7]. Two QTLs were identified for panicle number in the ratoon season in an F_2_ population [12].

Rice yield in the ratoon season largely depends on regenerated tiller numbers [13,14]. Till now, many genes have been cloned for rice tillering, such as *MOC1* [15], *LAX1* [16], *LAX2* [17], *D10* [18], *HTD1* [19], *D27* [20], *D53* [21], *OsTB1* [22], and *TAD1* [23]. Besides, a microRNA named *osa-MIR156f* was reported to regulate plant architecture and yield traits of rice both in the first season and ratoon season [13]. Overexpression of *osa-MIR156f* could down-regulate the expressions of *OsTB1* and *LAX1* and, finally, increase panicle number and yield in the ratoon season. Recently, Zhu et al. reported that expressions of *MOC1*, *LXA1*, and *LAX2* 72 hours after cutting panicles in the first season were significantly higher in varieties with higher ratooning ability, while *D10*, *HTD1*, *OsTB1*, and *TAD1* expressed higher in varieties with low ratooning ability [24]. The maximum tiller number in the ratoon season was significantly positively correlated with the expressions of *LAX2* and *MOC* and negatively correlated with the expressions of *D10*, *HTD1*, *OsTB1*, and *TAD1*.

In the present study, an introgression line (IL) population was developed from Minghui 63 (MH63) as the recipient parent and 02428 as the donor parent, and a high-density bin map was constructed using re-sequenced SNP data. Main-effect QTLs affecting ratoon-ability-related traits were identified in the first season and ratoon season. A pleiotropic QTL (*qRA5*) was validated using an IL (DQ146), and its genetic background effect was evaluated. Furthermore, *qRA5* was fine-mapped by a secondary population, derived from DQ146 and MH63. Our results will help us better understand the genetic basis of rice-ratooning ability and provide valuable gene resources for the breeding of high-yield ratoon rice variety.

## 2. Results

### 2.1. Bin Map of the IL Population

Among 58,936 polymorphic SNPs between the two parents, a total of 4568 bins were identified based on recombination sites in the IL population, covering 373.24 Mb of the Nipponbare genome. The length of the bin ranged from 30.00 kb to 1809.8 kb, with an average length of 80.71 kb.

**Figure 1:**
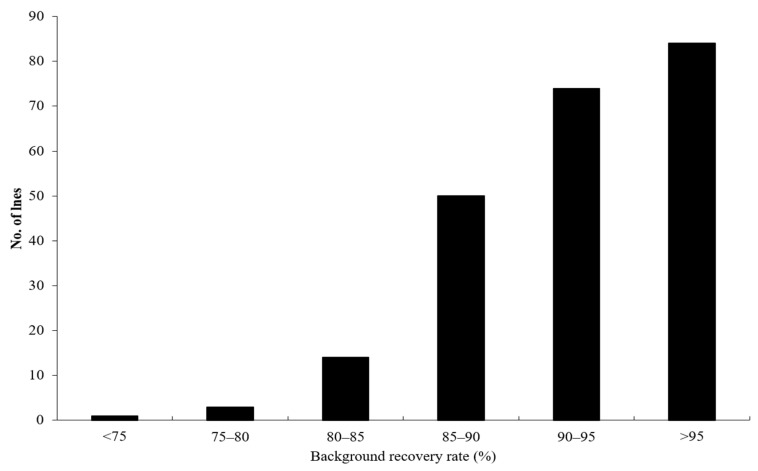
The genetic background recovery rate of the introgression line population.

The background recovery rates of ILs were demonstrated in Figure 1. The average background recovery rate of the IL population was 92.84%, with a range of 12.39–99.98%, and the background recovery rates of more than 70% ILs were above 90%, indicating that the genetic backgrounds of most of the ILs were similar with the recipient parent (MH63).

### 2.2. Performance of Ratooning Ability

As shown in Table 1, the two parents exhibited obvious differences in all traits, except GYR, in both years. MH63 had more tillers and panicles, and a higher yield in both the first and ratoon seasons in two years, resulting in MH63 having a higher ratooning ability than 02428. All traits showed continuous distributions and transgressive segregations in the IL population, suggesting that all nine traits were quantitative traits and controlled by multiple genes.

The correlations among different traits in the IL population were very similar between 2018 and 2019 (Table 2). In either the first season or ratoon season of each year, the maximum tiller number had a significantly positive correlation with the panicle number, and they were also significantly positively correlated with grain yield, suggesting that individuals with more tillers would have more panicles and higher grain yield. There were significantly positive correlations between two seasons within the same traits, indicating plants with more tiller and panicles as well as higher grain yield in the first season would have more tillers and panicles as well as higher grain yield in the ratoon season. The correlation coefficients between TNR and PNR, between TNR and GYR, and between PNR and GYR were all significant, indicating that varieties with higher TNR would have higher PNR and GYR. As expected, TNR, PNR, and GYR had significantly negative correlations with FTN, FPN, and FGY, respectively, in the first season, while all of them were significantly positively correlated with FTN, FPN, and FGY in the ratoon season, except between TNR and RGY in 2019, indicating that the ratooning ability was more correlated with the ratoon season than the first season.

### 2.3. QTL Mapping of Ratooning Ability

**Figure 2:**
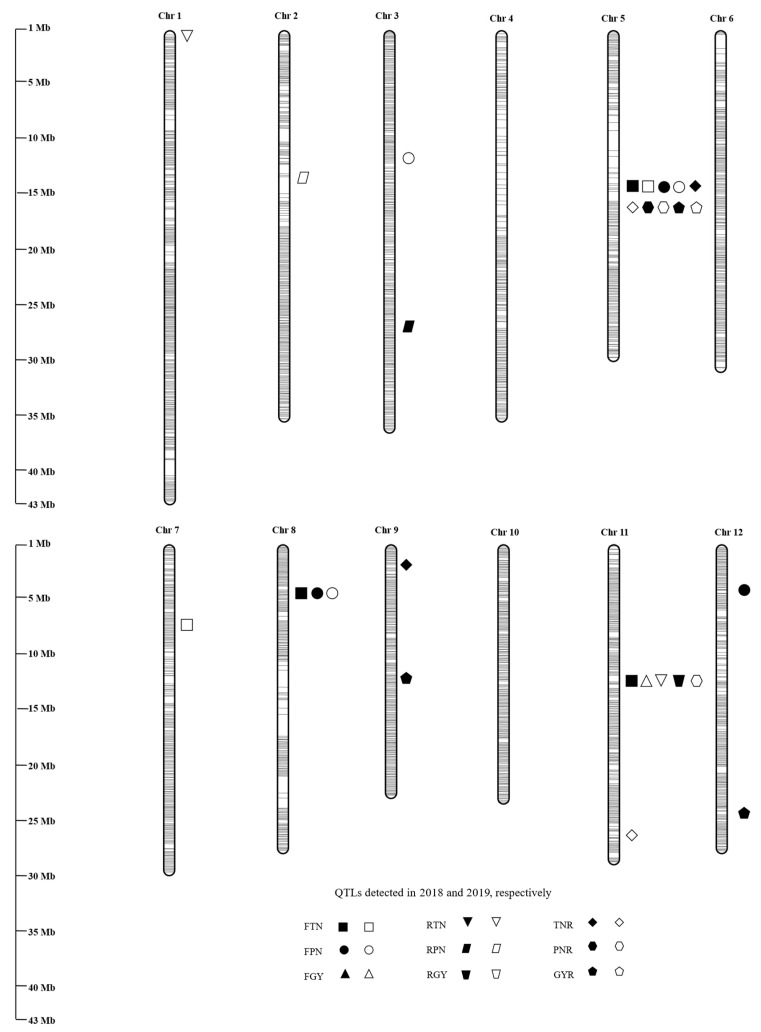
Genome distribution of M-QTLs detected for ratooning ability in the IL population derived from MH63 and 02428. FTN, maximum tiller number per plant in the first season; FPN, panicle number per plant in the first season; FGY, grain yield per plant in the first season; RTN, maximum tiller number per plant in the ratoon season; RPN, panicle number per plant in the ratoon season; RGY, grain yield per plant in the ratoon season; TNR, maximum tiller number ratio of RTN to FTN; PNR, panicle number ratio of RPN to FPN; GYR, grain yield ratio of RGY to FGY.

A total of 22 M-QTLs for nine ratooning-ability-related traits were identified on chromosomes 1–3, 5, 7–9, 11, and 12 in 2018 and 2019. Among them, six QTLs were detected in both years, and the others were identified in one year (Table 3; Figure 2).

In the first season, a total of 9 QTLs were identified, including four, four, and one QTLs for FTN, FPN, and FGY, respectively (Table 3; Figure 2). Among them, 3 QTLs (*qFTN8*, *qFTN11*, and *qFPN12*) were detected only in 2018, accounting for 8.69–16.71% of the phenotypic variations, while *qFTN7*, *qFPN3*, and *qFGY11* were identified only in 2019, explaining 8.11–10.66% of the phenotypic variations. Three QTLs (*qFTN5*, *qFPN5*, and *qFPN8*) were stably expressed in both years with mean phenotypic variation of 13.33%, 15.26%, and 7.76%, respectively. MH63 alleles at all QTLs increased traits.

Five QTLs were identified in the ratoon season, including two, two, and one QTLs for RTN, RPN, and RGY, respectively (Table 3; Figure 2). They were distributed on chromosomes 1–3 and 11. Among them, three QTLs (*qRTN11*, *qRPN3*, and *qRGY11*) were identified in 2018 and explained 3.26–9.52% of the phenotypic variations. The remaining two QTLs (*qRTN1* and *qRPN2*) were found only in 2019, accounting for 4.35% and 4.27% of phenotypic variation, respectively. MH63 alleles at all QTLs increased traits.

A total of eight QTLs were identified for ratooning ability, including three, three, and two QTLs for TNR, GYR, and PNR, respectively (Table 3; Figure 2). Among them, three QTLs (*qTNR5*, *qPNR5*, and *qGYR5*) were identified in both years with the mean phenotypic variations of 13.95%, 9.41%, and 11.59%. *qTNR9*, *qGYR9*, and *qGYR12* were expressed only in 2018 and accounted for phenotypic variation of 5.89–11.20%. The other two QTLs (*qTNR11* and *qPNR11*) were found only in 2019 and explained 14.00% and 9.73% of phenotypic variations. MH63 alleles were associated with increased trait values at *qTNR9*, *qTNR11*, *qGYR5*, and *qGYR12*, while associated with decreased trait values at the rest of the four QTLs.

Among the 22 QTLs detected in this study, three regions had pleiotropic effects on two or more traits (Table 3; Figure 2). For example, the region of 14.12–14.65 Mb on chromosome 5 harbored *qFTN5* for FTN, *qFPN5* for FPN, *qTNR5* for TNR, *qPNR5* for PNR, and *qGYR5* for GYR; the region of 4.64–5.76 Mb on chromosome 8 harbored *qFTN8* for FTN and *qFPN8* for FPN; and the 10.64–15.52 Mb region on chromosome 11 harbored *qFTN11* for FTN, *qFGY11* for FGY, *qRTN11* for RTN, *qRGY11* for RGY, and *qPNR11* for PNR.

### 2.4. Validation of qRA5

The region of 14.12–14.65 Mb on chromosome 5 had a pleiotropic effect on FTN, FPN, TNR, PNR, and GYR in both 2018 and 2019. Thus, it was called pleiotropic QTL *qRA5* (*ratooning ability 5*). An IL (DQ146) was selected and used for validation of the gene effect of *qRA5* (Table 4). DQ146 had 82.5% genome recovery of MH63 and did not carry any other M-QTL, except *qRA5* in the whole genome, so it could be considered a near-isogenic line of MH63. The FTN, FPN, and GYR of DQ146 were 18.50, 8.67, and 0.58, respectively, significantly lower than 21.53, 11.33, and 0.71 for MH63, while the TNR and PNR of DQ146 were 1.24 and 1.90, respectively, significantly higher than those of 0.95 and 1.65 of MH63. The above results indicated that *qRA5* was truly inherited and had pleiotropic effects on FTN, FPN, TNR, PNR, and GYR.

### 2.5. Genetic Background Effect on qRA5

To identify the genetic background effect on *qRA5*, one IL (DQ285) was also developed using 02428 as the recipient parent and MH63 as the donor parent, with a background recovery rate of 88.9%. It did not contain any other M-QTL, except *qRA5* in the whole genome, and could be also considered as a near-isogenic line of 02428. The FTN and FPN of DQ285 were significantly higher than those of 02428, while its TNR and PNR were significantly lower than those of 02428 (Table 5). The results suggested that *qRA5* was consistently expressed in *indica* (MH63) and *japonica* (02428) backgrounds, independent of genetic background.

### 2.6. Substitution Mapping of qRA5

**Figure 3:**
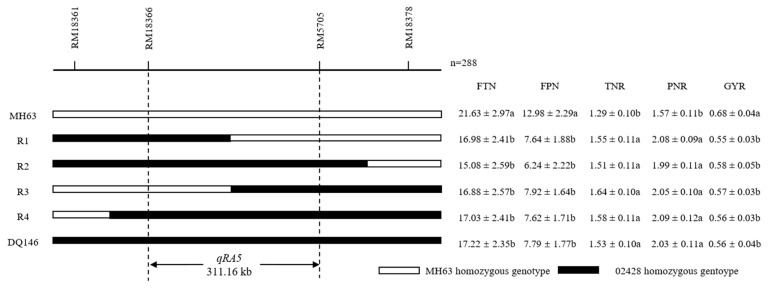
Substitution mapping of *qRA5*. Letter a or b represent significance between them at level of *p* < 0.05. FTN, maximum tiller number per plant in the first season; FPN, panicle number per plant in the first season; TNR, maximum tiller number ratio of RTN to FTN; PNR, panicle number ratio of RPN to FPN; GYR, grain yield ratio of RGY to FGY.

An F_2_ segregation population derived from DQ146 and MH63 was used for substitution mapping of *qRA5*. Among the 11 SSR markers in the region of *qRA5*, four markers (RM18361, RM18366, RM5705, and RM18378) had polymorphism between MH63 and 02428. Two hundred and eighty-eight individuals in the F_2_ segregation population were genotyped using the four polymorphic markers. Four recombinants were identified in the interval of RM18361-RM18378, and their homozygous progenies were used for phenotypic evaluation (Figure 3). Both R1 and R3 showed significantly lower FTN, FPN, and GYR as well as significantly higher TNR and PNR than those of MH63, suggesting *qRA5* was delimited in the region of approximately 311.16 kb, flanked by RM18366 and RM5705.

## 3. Discussion

Ratoon ability is a very important factor that influences yield in ratoon season, and it is controlled by multiple genes and largely affected by the environment. In the present study, a total of 22 QTLs were identified for nine ratooning-ability-related traits in an IL population derived from MH63 as the recipient parent and 02428 as the donor parent; some QTLs were located in the same or adjacent regions of QTLs [7,10,12], with genes [25,26,27] previously reported, as shown in Table 3. In particular, *qRA5* in the region of 14.12–14.65 Mb on chromosome 5 was located near *qETN5* [10], *qRA5* [10], and *qRAT5* [7]. Most previously reported QTLs were identified in large regions and have not been validated. Moreover, some previously cloned genes for tiller number or panicle number remain to be identified for their effects on ratoon ability. Allelic correspondence of the QTLs for ratooning ability, as identified in the present study along with previously reported ones, should be further investigated by fine-mapping and cloning. Besides, the only validated QTL, *qRAT5*, was substitution mapped between InDel 3 and RM249 in a large region of 5.29 Mb. In the present study, the *qRA5* was validated by an IL (DQ146) and has been fine-mapped by a substitution mapping approach in a smaller region, with 311.16 kb flanked by RM18366 and RM5705. There were nine newly identified QTLs (*qFTN8*, *qFPN3*, *qFPN8*, *qFPN12*, *qRTN1*, *qRPN1*, *qTNR9*, *qGYR9*, and *qGYR12*) in the present study. With increasing attention paid to this aspect, more consistent QTLs in different studies would be identified, and the genetic basis of ratoon ability would be clearly revealed.

There were 47 candidate genes in the region of *qRA5* (http://rice.plantbiology.msu.edu/; accessed on 1 July 2021). Among them, 26 genes encode transposon protein, 10 genes encode expressed protein, and 2 genes encode hypothetical protein. Besides, *LOC_Os05g24930* and *LOC_Os05g25060* encode PPR repeat-containing protein, *LOC_Os05g24684* encodes structural constituent of ribosome, *LOC_Os05g24760* encodes Harpin-induced protein 1 domain-containing protein, *LOC_Os05g24770* encodes reticulon domain-containing protein, *LOC_Os05g24780* encodes OsCML21-calmodulin-related calcium sensor protein, *LOC_Os05g24880* encodes dehydrogenase, *LOC_Os05g24890* encodes tetratricopeptide-like helical, and *LOC_Os05g24970* encodes LSM domain-containing protein. In previous studies, a lot of PPR proteins were reported to be associated with the biosynthesis of chloroplast [28,29,30], and many white or yellow color leaf mutants exhibited a decreased panicle number [31,32], so they may affect tiller number and ratooning ability. A gene (*OsALDH2B1*) encoding aldehyde dehydrogenase was reported to control tiller number [33], thus, genes encoding dehydrogenase may also control ratooning ability. Therefore, *LOC_Os05g24930*, *LOC_Os05g24880*, and *LOC_Os05g25060* were more likely the candidate genes. Among the three candidate genes, there was only one non-synonymous SNP (G/T), in the fourth exon of *LOC_Os05g25060*. The MH63 allele encodes Arg, while the 02428 allele encodes Met. Moreover, according to the CREP database (http://crep.ncpgr.cn/crep-cgi/home.pl; accessed on 1 March 2010), the *LOC_Os05g25060* was expressed highly in the stem during the tilling stage. Thus, *LOC_Os05g25060* was the most likely candidate gene of *qRA5*. Another region of 10.64–15.52 Mb on chromosome 11 is important, affecting FTN, FGY, RTN, RGY, and PNR, and involving grain yield both in the first season and ratoon season. Next, we will develop a large segregating population to fine-map the important gene for rice ratoon ability inside this region.

Increasing ratoon rice yield is a very important breeding goal, in the area where light and temperature are not enough for second season rice. However, the rice ratooning ability is a complex quantitative trait controlled by multiple genes and largely affected by the environment. Thus, identifying QTL and introgressing them into elite rice varieties is an effective way to improve ratooning ability. In many previous studies, the genetic background effect in QTL expressions was identified to be very large for appearance quality [34] and other traits. Besides, pleiotropic QTL also had a large value for improving ratooning ability, as breeders could only introduce the pleiotropic QTL instead of pyramiding multiple loci and save a lot of labor, money, and time. To date, many yield-related genes with pleiotropic effects on different traits have been identified and cloned, such as *Ghd7* [35] and *Ghd7.1* [36]. In the present study, three genomic regions, including 14.12–14.65 Mb on chromosome 5, 4.64–5.76 Mb on chromosome 8, and 10.64–15.52 Mb region on chromosome 11, simultaneously controlled two or more ratooning-ability-related traits and would be very valuable in molecular breeding for improving rice ratooning ability. Moreover, the region of 14.12–14.65 Mb on chromosome 5 was validated to be independent of genetic background. Although MH63 alleles decreased TNR and PNR in the region of 14.12–14.65 Mb on chromosome 5 and PNR in the region of 10.64–15.52 Mb region on chromosome 11, they could increase FTN, FPN, FGY, RGY, and GYR. The MH63 alleles of these three regions could be pyramided, simultaneously increase FTN and FPN, and, finally, increase grain yield in both the first and ratoon seasons. Besides, novel alleles increasing ratooning ability at important QTLs such as *qRA5* should be mined from rice germplasms in the future, enriching gene resources for the molecular breeding of high-yield ratoon rice varieties.

## 4. Materials and Methods

### 4.1. Development of IL Population

MH63 is the restorer parent of Shanyou 63, a widely adapted *indica* hybrid variety in China with high ratooning ability, while 02428 is a typical *japonica* variety, with wide compatibility and low ratooning ability. MH63 was used as a female parent to cross with 02428, F_1_ was backcrossed with MH63 to generate a BC_1_F_1_ population with about 80 plants, and BC_1_F_1_ individuals were randomly used as male parents to cross with MH63 to produce a BC_2_F_1_ population with about 200 individuals. Then, the BC_2_F_1_ progenies were successively self-crossed for 6 generations with no selection from BC_2_F_1_ to BC_2_F_7_ generation. Finally, a set of BC_2_F_7_ ILs population containing 226 lines was successfully developed.

### 4.2. Genotyping of the IL Population

Young leaves of about 20 individuals of each line and two parents were harvested, and DNA was extracted using the DNeasy Mini Kit (Qiagen). Their genomes were re-sequenced using an Illumina Genome Analyzer Ⅱx, as described by Huang et al. [37]; then, the genotypes of ILs were determined based on the SNPs generated by whole-genome sequencing. The whole genomes were obtained as 5,336,108,154 bp and 5,562,905,674 bp of data for MH63 and 02428, respectively, while 5,062,106,567 bp (96.57%) and 5,278,080,725 bp (94.03%) of consistent sequences were obtained and aligned to the Nipponbare sequence (IRGSP 1.0) [38]. There were 58,936 polymorphic SNPs between the two parents. These SNPs were used for constructing a bin map with 4568 bins, as described by Xie et al. [39]. A bin was defined as coverage of SNPs that have the same genotypes along a chromosome.

### 4.3. Field Experiment and Trait Evaluation

A total of 226 ILs and parents were grown in the spring to autumn seasons of 2018 and 2019 on the experimental farm of Yangtze University in Jingzhou (30.18° N, 112.15° E), China. In both years, all materials were sown on 20 March and transplanted on 25 April. Each line was planted in five rows with 10 plants in each row, at a spacing of 20 cm × 20 cm. Field experiments were conducted using a randomized complete block design with two replications. All field management followed local farmers’ practices.

At the tillering stage in the first season, ten individuals with uniform growth in the middle rows of each line were measured for their tiller numbers every week, to determine the maximum tiller number per plant (FTN). At the heading stage, heading dates were recorded. At the maturity stage, panicle number per plant (FPN) was investigated, then harvested 30 days after flowering. The yield of plots was measured and the grain yield per plant (FGY) was calculated by the ratio of the yield of a plot to plant number. The stubble of each plant was maintained at the height of about 40 cm, as described by He et al. [3].

In ratoon season, individuals measured in the first season of each line were measured for their tiller numbers every 3 days, to determine the maximum tiller number per plant (RTN). Heading date and panicle number per plant (RPN) were investigated at the heading and maturity stages, respectively. Then, all measured plants were harvested about 25 days after flowering. The yield of plots was measured, and the grain yield per plant (RGY) was determined by the ratio of the yield of a plot to plant number.

The ratooning ability was measured as three traits: tiller number ratio (TNR), determined by the ratio of RTN to FTN; panicle number ratio (PNR), determined by the ratio of RPN to FPN; and grain yield ratio (GYR), determined by the ratio of RGY to FGY.

### 4.4. Data Analysis and QTL Mapping

Statistical description and correlations among different traits of the IL population and the difference of traits between ILs were analyzed by Statistica 5.5 [34]. Main-effect QTL was identified, using the inclusive interval mapping (ICIM) function with the bi-parent population (BIP) module in IciMapping 4.0 [40], and LOD thresholds were set as 3.0.

### 4.5. Validation of qRA5

*qRA5* affecting five ratooning-ability-related traits was identified in the region of 14.12–14.65 Mb on chromosome 5 in both years. To confirm *qRA5*, one IL (DQ146) was selected from the IL population because it carried only one QTL (*qRA5*), without genetic noise resulting from any other QTL for ratooning-ability-related traits and a higher recovery rate of the MH63 genome. DQ146 and MH63 were planted in the spring to autumn seasons of 2020 on the experimental farm of Yangtze University in Jingzhou, China, with eight rows and 10 individuals per row. Twenty individuals with uniform growth in the middle rows of each line were selected, and all their ratooning-ability-related traits (FTN, FPN, FGY, RTN, RPN, and RGY) were evaluated; thus, TNR, PNR, and GYR were calculated, following the method described above. Differences were tested for TNR, PNR, and GYR between DQ146 and MH63 using the Ducan t-test at the threshold of *p* ≤ 0.05.

### 4.6. Detection of Genetic Background Effect on qRA5

One IL (DQ285) with MH63 allele of *qRA5* at 02428 background was developed [34]. It was also planted in the spring to autumn seasons of 2020 on the experimental farm of Yangtze University in Jingzhou, China, with eight rows and 10 individuals per row. About 20 individuals with uniform growth in the middle rows were selected and all their ratooning-ability-related traits were evaluated following the method as validation of *qRA5*. Finally, the differences between DQ285 and 02428 were tested using the Ducan t-test at the threshold of *p* ≤ 0.05.

### 4.7. Substitution Mapping of qRA5

DQ146 was crossed with MH63 and generated an F_2_ segregating population. The F_2_ population comprising 288 plants was grown in the summer season of 2020 on the experimental farm of Yangtze University in Jingzhou, China. Eleven simple sequence repeat (SSR) markers (RM18359, RM18360, RM18361, RM7449, RM18366, RM18368, RM18371, RM18374, RM5705, RM18376, and RM18378) in the region of 14.12–14.65 Mb on chromosome 5, according to the Gramene database (https://www.gramene.org/markers; accessed on 1 January 2005), were used to evaluate the genotypic polymorphism between MH63 and 02428. PCR and electrophoresis followed the method described by Jiang et al. [41]. PCR was conducted in a 20 μg reaction mixture containing 50 ng genomic DNA, 10 mM Tris-HCl, 50 mM KCl, 1.5 mM MgCl_2_, 2 mM dNTP, 0.1 mM of each primer, and 1 unit Taq polymerase. The template PCR was initially denatured at 95 ℃ for 5 min, followed by 35 cycles performed at 95 ℃ for 30 s, 55 ℃ for 30 s, and 72 ℃ for 1 min, and, then, a final extension at 72 ℃ for 5 min. The PCR products were then electrophoretically resolved on a 4% polyacrylamide gel. Recombinant individuals with different genotypes of the flanking markers were selected by the polymorphic markers flanking the QTL. About 100 progeny plants of each recombinant were planted in the spring to autumn seasons of 2021 on the experimental farm of Yangtze University in Jingzhou, China. All of them were genotyped by the polymorphic markers to select the homozygous recombinant plants. About 20 homozygous progenies of each recombinant were evaluated for their ratooning ability, following the method as validation of *qRA5*. MH63 and DQ146 were used as the control. They were tested for the differences between homozygous recombinants and MH63, using the Duncan t-test at the threshold of *p* ≤ 0.05.

## 5. Conclusions

QTL mapping affecting nine ratoon-ability-related traits was performed using a bin map containing 4568 bins in an IL population derived from MH63 as the recipient parent and 02428 as the donor parent. Nine ratoon-ability-related traits showed continuous distributions and transgressive segregations. A total of 22 main-effect QTLs were identified for nine ratoon-ability-related traits. Three genomic regions, including 14.12–14.65 Mb on chromosome 5, 4.64–5.76 Mb on chromosome 8, and 10.64–15.52 Mb on chromosome 11, were identified to simultaneously control two or more ratooning-ability-related traits. Of them, a pleiotropic QTL *qRA5* was validated, independently of genetic background, and mapped into a 311.16 kb region flanked by RM18366 and RM5705. MH63 alleles at QTLs in the above three regions could be used for molecular breeding to improve rice ratooning ability.

## Figures and Tables

**Table 1:** Performance of ratooning-ability-related traits of the parents and their derived IL population.

Trait	Year	MH63	02428	IL Population			
				Mean ± SD	Maximum	Minimum	CV (%)
FTN	2018	24.52	9.03 **	21.18 ± 4.29	30.33	5.25	20.24
	2019	30.21	11.33 **	28.69 ± 5.26	42.17	4.08	18.33
FPN	2018	16.64	4.84 **	12.48 ± 2.92	19.17	3.83	23.39
	2019	17.08	4.99 **	16.79 ± 3.63	21.65	2.99	21.62
FGY (g)	2018	23.05	7.38 **	18.69 ± 6.21	34.66	2.49	33.20
	2019	25.08	8.11 **	21.09 ± 8.81	42.66	4.07	41.77
RTN	2018	33.69	7.86 **	23.50 ± 6.82	46.17	4.33	29.03
	2019	36.60	9.08 **	27.88 ± 8.62	50.35	5.67	30.92
RPN	2018	22.27	3.94 **	13.07 ± 4.95	26.83	2.17	37.92
	2019	23.79	4.03 **	13.85 ± 5.02	29.91	3.39	36.25
RGY (g)	2018	11.05	2.43 **	5.62 ± 2.68	16.60	0.30	47.63
	2019	10.21	3.08 **	7.04 ± 3.01	17.84	0.26	42.76
TNR	2018	1.41	0.81 **	1.12 ± 0.28	2.30	0.27	24.64
	2019	1.28	0.78 **	1.05 ± 0.21	1.93	0.19	20.00
PNR	2018	1.33	0.88 *	1.05 ± 0.32	1.83	0.27	30.39
	2019	1.33	0.83 **	0.93 ± 0.29	1.99	0.33	31.18
GYR	2018	0.48	0.33	0.32 ± 0.17	1.54	0.06	53.97
	2019	0.44	0.35	0.33 ± 0.13	1.89	0.09	39.39

Note: *, ** represent significant levels of *p* < 0.05 and 0.01, respectively; FTN, maximum tiller number per plant in the first season; FPN, panicle number per plant in the first season; FGY, grain yield per plant in the first season; RTN, maximum tiller number per plant in the ratoon season; RPN, panicle number per plant in the ratoon season; RGY, grain yield per plant in the ratoon season; TNR, maximum tiller number ratio of RTN to FTN; PNR, panicle number ratio of RPN to FPN; GYR, grain yield ratio of RGY to FGY.

**Table 2:** Correlation coefficients of ratooning-ability-related traits in the IL population derived from MH63 and 02428.

Trait	FTN	FPN	FGY	RTN	RPN	RGY	TNR	PNR	GYR
FTN		0.68 **	0.32 **	0.44 **	0.39 **	0.11	−0.31 **	−0.04	0.01
FPN	0.74 **		0.28 **	0.75 **	0.70 **	0.15 *	0.07	−0.25 **	0.04
FGY	0.26 **	0.22 **		0.09	0.18 **	0.31 **	−0.37 **	0.08	−0.42 **
RTN	0.58 **	0.69 **	−0.06		0.85 **	0.32 **	0.57 **	0.30 **	0.19 **
RPN	0.49 **	0.62 **	0.15 *	0.72 **		0.66 **	0.48 **	0.81 **	0.29 **
RGY	0.17 **	0.19 **	0.40 **	0.24 **	0.51 **		0.07	0.58 **	0.77 **
TNR	−0.23 **	0.13	−0.28 **	0.63 **	0.37 **	0.14 *		0.30 **	0.42 **
PNR	0.07	−0.19 **	−0.01	0.36 **	0.77 **	0.47 **	0.34 **		0.58 **
GYR	−0.04	−0.02	−0.31 **	0.26 **	0.36 **	0.68 **	0.35 **	0.48 **	

Note: Data under and above the diagonal are the correlation coefficients in 2018 and 2019, respectively; *, ** represent significant levels of *p* < 0.05 and 0.01, respectively; FTN, maximum tiller number per plant in the first season; FPN, panicle number per plant in the first season; FGY, grain yield per plant in the first season; RTN, maximum tiller number per plant in the ratoon season; RPN, panicle number per plant in the ratoon season; RGY, grain yield per plant in the ratoon season; TNR, maximum tiller number ratio of RTN to FTN; PNR, panicle number ratio of RPN to FPN; GYR, grain yield ratio of RGY to FGY.

**Table 3:** Main-effect QTLs for ratooning-ability-related traits in the IL population derived from MH63 and 02428.

Trait ^1^	QTL	Year	Chr.	Position (Mb)	LOD	A ^2^	*R^2^*(%) ^3^	QTLs or GENES Previously Reported
FTN	*qFTN5*	2018	5	14.12–14.65	17.47	−0.98	16.45	*qENT5, qRA5, qRAT5* [7,10]
		2019	5	14.12–14.65	10.88	−0.75	10.21	
	*qFTN7*	2019	7	2.48–3.61	4.62	−0.24	9.95	*RLB/OsH15* [25]
	*qFTN8*	2018	8	4.64–5.76	12.54	−0.67	11.63	
	*qFTN11*	2018	11	10.64–15.52	15.99	−0.53	16.71	*qRa11* [12]
FPN	*qFPN3*	2019	3	11.45–12.51	3.47	−0.28	8.11	*SLR1* [26]
	*qFPN5*	2018	5	14.12–14.65	16.26	−0.60	16.30	*qENT5, qRA5, qRAT5* [7,10]
		2019	5	14.12–14.65	10.37	−0.39	14.21	
	*qFPN8*	2018	8	4.64–5.76	10.30	−0.24	10.21	
		2019	8	4.64–5.76	5.00	−0.15	5.31	
	*qFPN12*	2018	12	3.88–5.51	4.54	−0.11	8.69	
FGY	*qFGY11*	2019	11	10.64–15.52	3.66	−1.91	10.66	*qRa11* [12]
RTN	*qRTN1*	2019	1	0–1.83	3.21	−0.09	4.35	
	*qRTN11*	2018	11	10.64–15.52	3.78	−0.18	9.52	*qRa11* [12]
RPN	*qRPN2*	2019	2	13.31–15.61	3.87	−0.10	4.27	
	*qRPN3*	2018	3	27.43–27.69	4.88	−0.27	3.26	
RGY	*qRGY11*	2018	11	10.64–15.52	4.91	−1.23	5.21	*qRa11* [12]
TNR	*qTNR5*	2018	5	14.12–14.65	29.56	0.16	18.63	*qENT5, qRA5, qRAT5* [7,10]
		2019	5	14.12–14.65	10.33	0.09	9.27	
	*qTNR9*	2018	9	0.87–4.04	17.20	−0.07	11.20	
	*qTNR11*	2019	11	25.90–27.71	3.57	−0.12	14.00	*MHZ5* [27]
PNR	*qPNR5*	2018	5	14.12–14.65	19.56	0.19	10.25	*qENT5, qRA5, qRAT5* [7,10]
		2019	5	14.12–14.65	8.66	0.13	8.57	
	*qPNR11*	2019	11	10.64–15.52	3.81	0.05	9.73	*qRa11* [12]
GYR	*qGYR5*	2018	5	14.12–14.65	28.60	−0.03	13.22	*qENT5, qRA5, qRAT5* [7,10]
		2019	5	14.12–14.65	8.54	−0.05	9.96	
	*qGYR9*	2018	9	11.77–14.01	31.72	0.05	10.95	*qRa11* [12]
	*qGYR12*	2018	12	24.68–25.37	23.88	−0.04	5.89	

^1^ FTN, maximum tiller number per plant in the first season; FPN, panicle number per plant in the first season; FGY, grain yield per plant in the first season; RTN, maximum tiller number per plant in the ratoon season; RPN, panicle number per plant in the ratoon season; RGY, grain yield per plant in the ratoon season; TNR, maximum tiller number ratio of RTN to FTN; PNR, panicle number ratio of RPN to FPN; GYR, grain yield ratio of RGY to FGY; ^2^ addictive effect, which was estimated by the substitution of the MH63 allele by 02428 allele; ^3^ *R^2^*, phenotypic variation explained by QTL.

**Table 4:** Comparison of ratooning-ability-related traits between DQ146 and Minghui 63.

Trait	MH63	DQ146
FTN	21.53 ± 3.21	18.50 ± 2.33 *
FPN	11.33 ± 2.33	8.67 ± 1.88 *
FGY	23.34 ± 4.52	24.45 ± 3.48
RTN	20.50 ± 3.26	23.00 ± 3.43
RPN	18.67 ± 3.01	16.67 ± 2.34
RGY	16.58 ± 2.99	14.27 ± 3.23
TNR	0.95 ± 0.13	1.24 ± 0.11 **
PNR	1.65 ± 0.14	1.90 ± 0.09 **
GYR	0.71 ± 0.09	0.58 ± 0.03 *

Note: *, ** represent significant levels of *p* < 0.05 and 0.01, respectively; FTN, maximum tiller number per plant in the first season; FPN, panicle number per plant in the first season; FGY, grain yield per plant in the first season; RTN, maximum tiller number per plant in the ratoon season; RPN, panicle number per plant in the ratoon season; RGY, grain yield per plant in the ratoon season; TNR, maximum tiller number ratio of RTN to FTN; PNR, panicle number ratio of RPN to FPN; GYR, grain yield ratio of RGY to FGY.

**Table 5:** Comparison of ratooning-ability-related traits between DQ285 at 02428 background and 02428.

Trait	02428	DQ285
FTN	8.83 ± 1.95	11.46 ± 1.89 **
FPN	5.17 ± 1.53	9.54 ± 1.66 **
FGY	2.49 ± 2.21	4.32 ± 2.40
RTN	10.25 ± 1.68	8.33 ± 1.54
RPN	5.50 ± 1.55	6.17 ± 1.73
RGY	0.77 ± 0.31	1.63 ± 0.72
TNR	1.16 ± 0.22	0.73 ± 0.25 *
PNR	1.06 ± 0.19	0.65 ± 0.24 **
GYR	0.31 ± 0.09	0.38 ± 0.11

Note: *, ** represent significant levels of *p* < 0.05 and 0.01, respectively; FTN, maximum tiller number per plant in the first season; FPN, panicle number per plant in the first season; FGY, grain yield per plant in the first season; RTN, maximum tiller number per plant in the ratoon season; RPN, panicle number per plant in the ratoon season; RGY, grain yield per plant in the ratoon season; TNR, maximum tiller number ratio of RTN to FTN; PNR, panicle number ratio of RPN to FPN; GYR, grain yield ratio of RGY to FGY.

## Abbreviations

FTN	maximum tiller number per plant in the first season
FPN	panicle number per plant in the first season
FGY	grain yield per plant in the first season
RTN	maximum tiller number per plant in the ratoon season
RPN	panicle number per plant in the ratoon season
RGY	grain yield per plant in the ratoon season
TNR	maximum tiller number ratio of RTN to FTN
PNR	panicle number ratio of RPN to FPN
GYR	grain yield ratio of RGY to FGY
IL	introgression line
QTL	quantitative trait locus
M-QTL	Main-effect quantitative trait locus
MH63	Minghui 63
SSR	simple sequence repeat
